# Pathophysiology and management of liver cirrhosis: from portal hypertension to acute-on-chronic liver failure

**DOI:** 10.3389/fmed.2023.1060073

**Published:** 2023-06-15

**Authors:** Rakesh Kumar Jagdish, Akash Roy, Karan Kumar, Madhumita Premkumar, Mithun Sharma, Padaki Nagaraja Rao, Duvvur Nageshwar Reddy, Anand V. Kulkarni

**Affiliations:** ^1^Department of Hepatology, Gastroenterology and Liver Transplant Medicine, Metro Hospital, Noida, India; ^2^Department of Gastroenterology, Institute of Gastrosciences and Liver Transplantation, Apollo Hospitals, Kolkata, India; ^3^Department of Hepatology, Mahatma Gandhi Medical College and Hospital, Jaipur, India; ^4^Department of Hepatology, Post Graduate Institute of Medical Education and Research (PGIMER), Chandigarh, India; ^5^Department of Hepatology, Asian Institute of Gastroenterology (AIG) Hospitals, Hyderabad, India

**Keywords:** portal hypertension, liver cirrhosis, HVPG, acute-on-chronic liver failure, chronic liver disease

## Abstract

Cirrhosis transcends various progressive stages from compensation to decompensation driven by the severity of portal hypertension. The downstream effect of increasing portal hypertension severity leads to various pathophysiological pathways, which result in the cardinal complications of cirrhosis, including ascites, variceal hemorrhage, and hepatic encephalopathy. Additionally, the severity of portal hypertension is the central driver for further advanced complications of hyperdynamic circulation, hepatorenal syndrome, and cirrhotic cardiomyopathy. The management of these individual complications has specific nuances which have undergone significant developments. In contrast to the classical natural history of cirrhosis and its complications which follows an insidious trajectory, acute-on-chronic failure (ACLF) leads to a rapidly downhill course with high short-term mortality unless intervened at the early stages. The management of ACLF involves specific interventions, which have quickly evolved in recent years. In this review, we focus on complications of portal hypertension and delve into an approach toward ACLF.

## 1. Introduction

Cirrhosis is a major cause of morbimortality, constituting around 2.4% of global deaths (1). The natural history of cirrhosis has a progressive and dynamic course transitioning from a relatively stable state of compensated cirrhosis to an advanced stage of decompensated cirrhosis (2). Central to the dynamics of the transition is the degree of portal hypertension (PH) which serves as the primary driver of complications like the development of varices, ascites, renal dysfunction, hepatic encephalopathy (HE), hyperdynamic circulation, and cardiomyopathy (3, 4). While on the one hand, the stagewise progression of cirrhosis with worsening of PH delineates the conventional natural history of cirrhosis, another distinct syndrome marked by an acute deterioration of liver function with or without extrahepatic organ failures known as acute-on-chronic liver failure (ACLF) has opened up newer paradigms in PH over the last decade (5). This review explores newer insights into the pathophysiology of PH in cirrhosis and ACLF.

## 2. Basic pathophysiological mechanisms of development of PH

**Figure 1:**
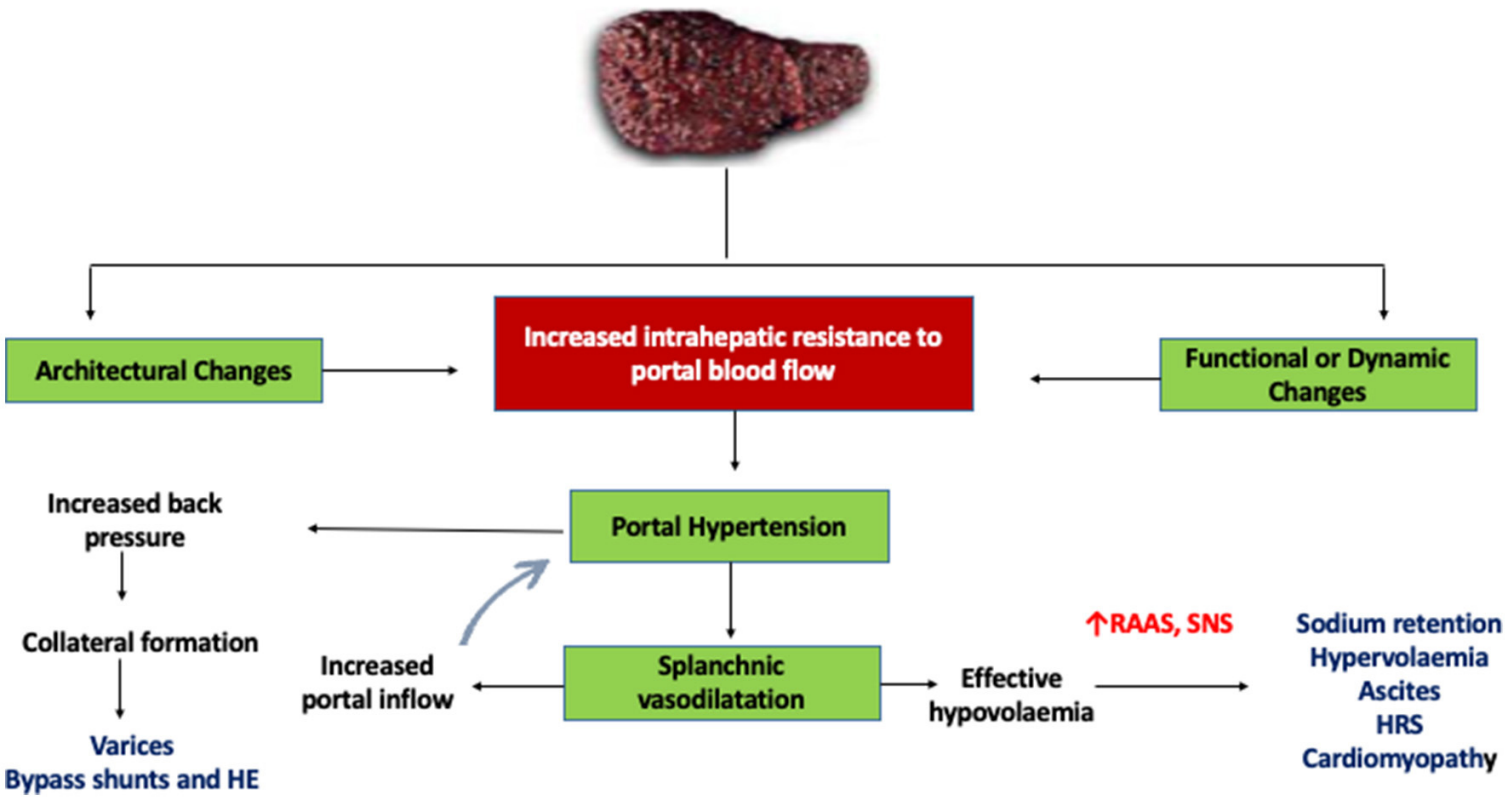
Mechanism of portal hypertension in cirrhosis. HE, hepatic encephalopathy; RAAS, renin-angiotensin-aldosterone system; SNS, sympathetic nervous system; HRS, hepatorenal syndrome.

**Figure 2:**
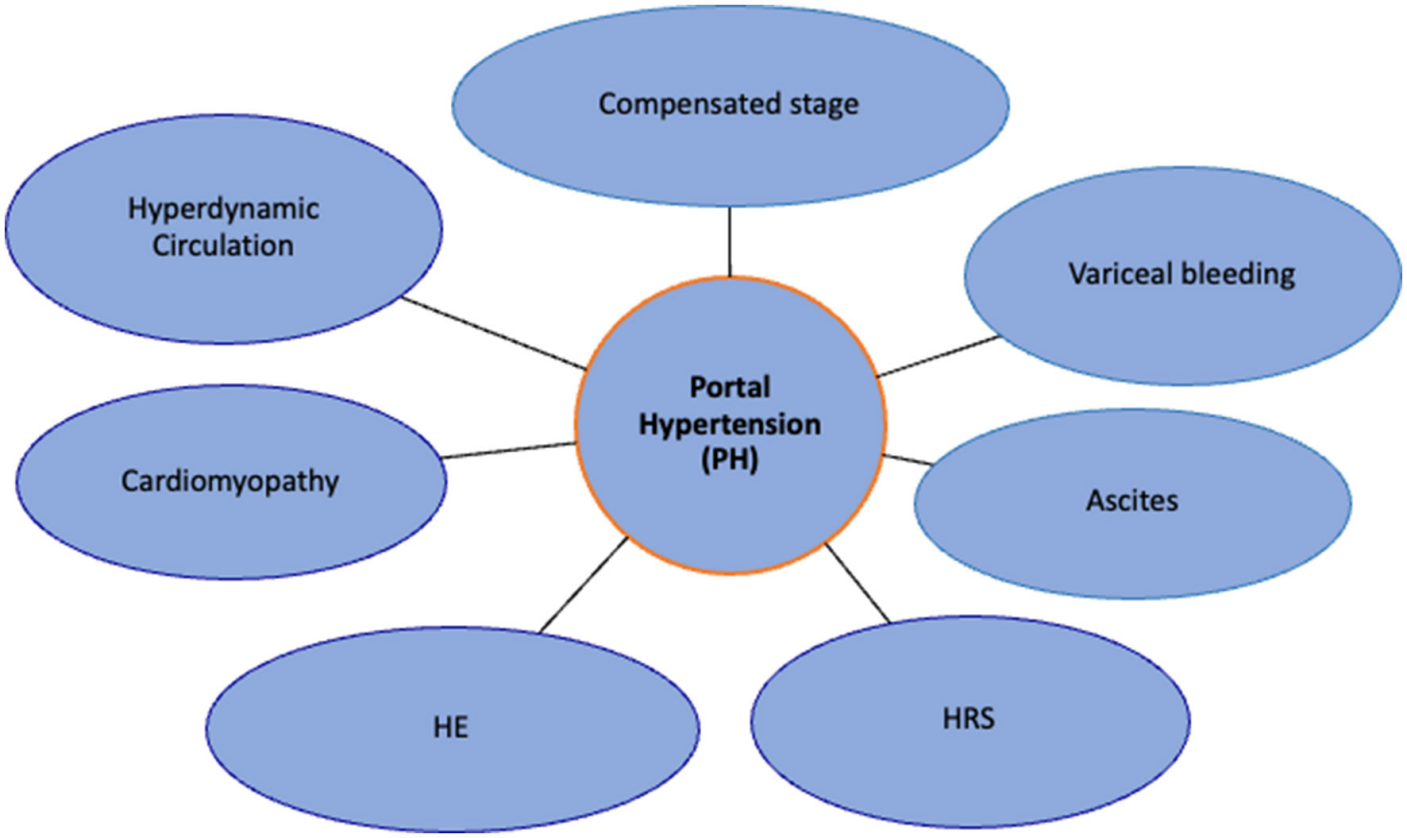
Effects of portal hypertension: migration from compensated stage to decompensated stage. HE, hepatic encephalopathy; HRS, hepatorenal syndrome.

Central to the development of PH is the occurrence of resistance at any point in the portal venous system, leading to the effect of a pressure gradient. In patients with cirrhosis, this resistance level is at the level of hepatic sinusoids, which arises from a combination of structural (fibrosis, nodule formation) and functional alterations (6). The static or architectural changes behind the development of PH are driven by alterations in the interplay between hepatic stellate cells (HSCs) and liver sinusoidal endothelial cells (LSECs). In response to any injury or insult, HSCs are activated and lead to extracellular matrix formation and fibrogenesis, while LSECs undergo a phenotypic remodeling leading to capillarization of the sinusoids, thereby increasing intrahepatic resistance. Coupled with this, a dynamic component arising from myofibroblast contraction and decreased vasodilators like nitric oxide further accentuate the resistance pathway (6, 7). These two fundamental mechanisms lead to the progressive development of PH, leading to splanchnic vasodilation, neurohormonal disturbances, systemic vasodilatation, decreased mean arterial pressures (MAP), and an overall hyperdynamic state (8) (Figure 1). In combination with these, gut microbial alterations, increased intestinal permeability, and systemic inflammation act as both precipitants and perpetrators of worsening PH and further downstream complications (8) (Figure 2). In the following sections, we elaborate on the individual consequences of PH and their management.

## 3. Variceal hemorrhage

### 3.1. Development of varices and importance of hepatic venous pressure gradient

Resistance to portal blood flow and increased portal venous blood inflow result in the reversal of flow and formation of alternate blood flow channels between the portal and systemic circulation, which result in varices. The development of varices acts as a surrogate marker of PH and signifies clinically significant portal hypertension (CSPH). HVPG is the closest surrogate marker of actual portal pressure and PH, with the presence of PH being defined as an HVPG > 5 mm Hg, while a value of >10 mmHg signifies CSPH (9) (Table 1). In patients with VH, an HVPG > 20 mmHg (measured within 24 h after admission) is the best predictor of a poor outcome. A reduction in the HVPG < 12 mm Hg or a reduction of more than 20% from the baseline value has been associated with a decreased risk of VH and improved survival (10). HVPG > 20 mm Hg has been associated with a 5.21-fold likelihood of rebleeding, and reducing HVPG below this threshold using a vasoactive drug improves outcomes. Patients with HVPG > 20 mmHg or < 10% decline in HVPG (non-responders) on vasoactive medications increases the risk of rebleeding and have higher mortality (10). All patients presenting with VH should ideally undergo HVPG measurement, although access to the procedure at all centers is limited (11, 12). Patients with VH who have an HVPG > 20 mmHg should be evaluated for an early transjugular portosystemic shunt (TIPSS) (13).

**Table 1:** Hepatic venous pressure gradient and esophageal varices.

**Event**	**HVPG (mm of Hg)**
Formation of varices (CSPH)	>10
Bleeding from varices	>12
Relatively no chances of re-bleed	< 16
Higher chances of re-bleed	>20
Early TIPS	>20

CSPH, clinically significant portal hypertension.

### 3.2. Risk factors for VH and risks associated with re-bleeding

VH from esophageal varices or gastric varices can result in high mortality (10–20% at 6 weeks) (3, 14). Other rare ectopic sites for VH (< 5% of VH) are the rectum, duodenum, and post-surgical stomas. There are multiple risk factors for VH, including the larger size of varices (>5 mm), higher HVPG, higher grade of the child class, presence of red color signs (RCS) markings, active alcohol consumption, and presence of sepsis. There are also certain high-risk factors for re-bleeding, including a pressure gradient measured within 24 h of bleeding more than 20 mmHg, presence of large varices, age ≥ 60 years, renal failure, and severe initial bleeding (on admission, hemoglobin < 8 g/dL) (11, 15).

### 3.3. Management of acute variceal bleeding

The management consists of controlling acute bleeding to prevent death and prevention of re-bleeding. Hemodynamic resuscitation is the initial treatment considering patient age, co-morbidities, ongoing blood loss, hemodynamic status, and other parameters. Fluid resuscitation should be cautious and restrictive to keep hemoglobin between 7 and 9 gm/dl, as overaggressive resuscitation can worsen PH and bleeding (16). INR-based corrections with fresh frozen plasma, factor VII transfusion, platelet, cryoprecipitate, or other blood products are not warranted (17, 18). Moreover, overzealous use of these products can be harmful due to the increase in PH due to volume overload or transfusion-related lung injury (14, 19). After gastrointestinal (GI) bleeding, blood acts as a culture media to grow infections; therefore, adequate purging should be done to prevent post-bleed sepsis, HE, ascites, or other complications of PH. Post-bleed sepsis can increase mortality; thus mandating the use of antibiotics during bleeding events as per local antibiograms. Currently, third-generation cephalosporins are recommended **(**ceftriaxone 1 gm IV every 24 h for 7 days) (20, 21). Vasoconstrictors should be started as early as possible in VH, along with proton-pump inhibitors. Vasoconstrictors should be continued for at least 2–5 days (15). Somatostatin, octreotide, and terlipressin are the recommended agents with comparable efficacy and safety (22).

**Figure 3:**
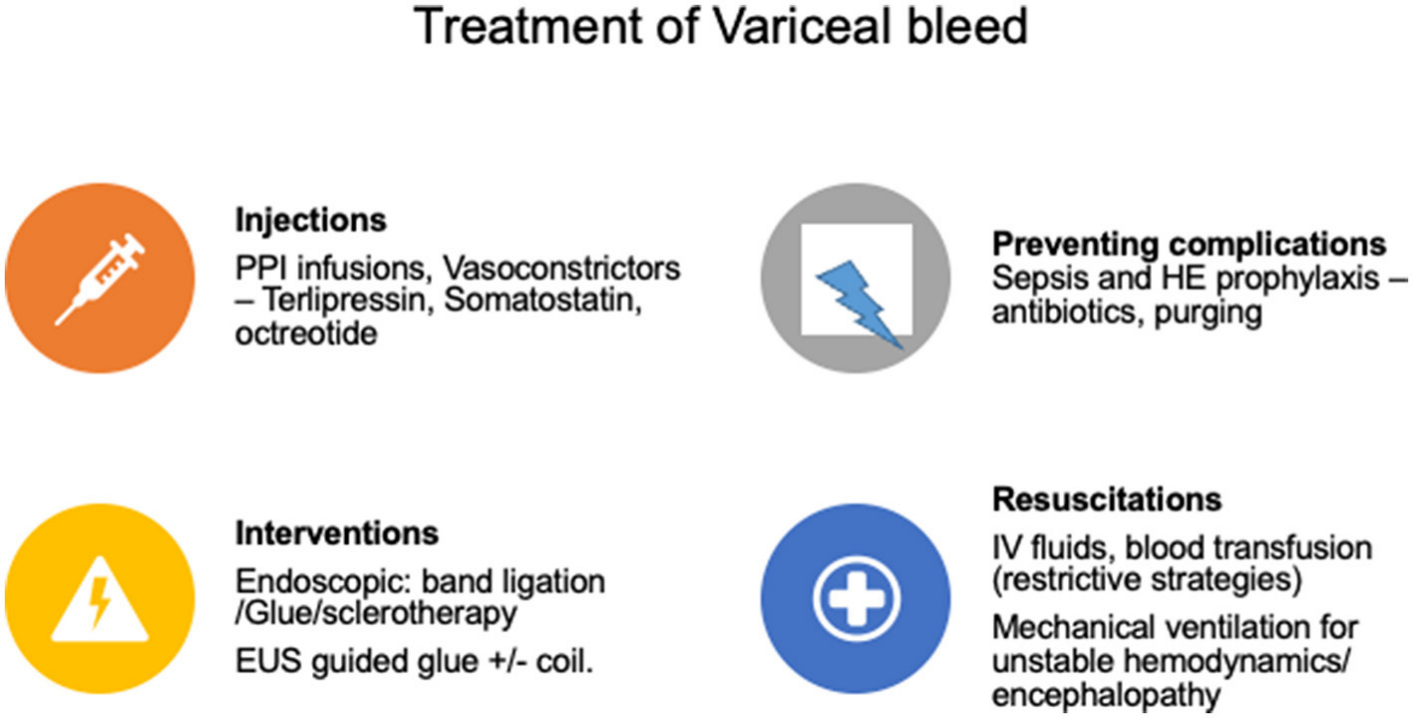
Treatment of variceal hemorrhage. HE, hepatic encephalopathy; IV, intravenous.

Endoscopy-based endotherapy is definitive in managing VH and should be done within 12 h after hemodynamic resuscitation (23). Prokinetics (intravenous erythromycin) and anti-emetics should be given before the endoscopy for better visualization (24). Patients with altered mentation, severe sepsis, shock, and acidosis should be electively intubated before endoscopy. Endoscopic band ligation (EBL) is the definitive therapy for esophageal varices and gastro-esophageal varices (GOV) type 1. Endoscopic glue injection with cyanoacrylate glue remains the most used therapy for treating bleeding from isolated gastric varices (IGV) and GOV type 2 (Figure 3). Tamponade with Sengastaken–Blakemore (SB tube) or Minnesota tube is usually considered a salvage modality in cases of refractory bleeding, often serving as a bridge to more definitive therapy such as TIPSS. The role of TIPSS in VH has been advocated as a pre-emptive modality (pre-emptive TIPSS) and a salvage modality (rescue TIPSS) (25). After stabilization, imaging studies (ultrasonography/computed tomographic scan) to rule out acute causes of PH like portal vein thrombosis (PVT) and hepatocellular carcinoma (HCC) should be performed (26).

### 3.4. Newer perspectives

An emerging concept proposed is identifying risk factors and possible avoidance of antibiotics in patients with well-preserved liver functions presenting with VH, however, prospective validation is needed (27, 28). Although the model for end-stage liver disease (MELD) is reasonable in predicting outcomes of patients with VH, a recent study reported MELD-Lactate to be superior in predicting mortality after VH (29, 30).

### 3.5. Primary prophylaxis of VH

Non-selective beta-blockers (NSBBs) or EBL are the treatments of choice to prevent VH (31). The use of NSBBs in PH is well-studied and has a pleiotropic mechanism. In addition to being economical to use, recent studies have demonstrated their pleiotropic effects, like preventing bacterial translocation, antioxidant properties, containing further non-bleed decompensations, and portal hypertensive gastropathy progression, as well as improving survival in ACLF (32–35). Adding another rate-controlling agent, ivabradine, to NSBB has shown some promising results, achieving better hemodynamics, reducing the incidence of acute kidney injury (AKI) and HE, and achieving a target heart rate (36). However, external validation of this merits consideration.

Gastric variceal bleeds account for ~20% of total variceal bleeds, are more profuse, are predominantly flow-related rather than pressure-related, and have higher mortality. Primary prophylaxis for GOV-1 is similar to EV: with either NSBB or balloon/coil/plug-assisted retrograde transvenous obliteration (BRTO/PARTO/CARTO) of gastrorenal/lienorenal shunt for patients with a history of HE. For patients with high-risk (size > 20 mm or severe PHG or MELD > 17) GOV2/IGV1, it may be preferable to perform CARTO/PARTO if there is a gastro renal shunt. Otherwise, an endoscopic ultrasonography-guided coil with or without NSBB or prophylactic cyanoacrylate injection is suggested in addition to NSBB. For patients with low-risk GOV2/IGV1 (< 10 mm), NSBBs would be sufficient (37).

### 3.6. Newer perspectives

Emerging data have frequently advocated BRTO to be effective in managing gastric variceal bleeding. A recent Korean study shows that BRTO and endoscopic obliteration are equivalent in preventing gastric variceal bleeds compared to placebo (38). This retrospective study needs further validation.

### 3.7. Secondary prophylaxis

Propranolol first demonstrated its effectiveness in preventing recurrent esophageal variceal bleeding in 1980 (39). Later, carvedilol was introduced, which has a better profile than propranolol. The addition of carvedilol to EBL than propranolol to EBL can lead to better HVPG response (40). NSBB reduces and prevents death while waiting for liver transplantation (LT) in patients with refractory ascites (RA) and/or VH, but controversies in advanced decompensated patients with ascites remain (41). TIPSS is traditionally performed for patients with refractory bleeding who fail EBL + NSBB. A recent study on early TIPSS (stent placement within 5 days of variceal bleed) has shown significant mortality benefits with a substantial reduction in the recurrence of variceal bleeding without increasing the risk of HE (42). In gastric variceal bleeding, TIPSS has been shown to prevent gastric variceal re-bleeding in patients with high HVPG (43, 44). BRTO, where the target flow is selectively occluded, is more effective than TIPSS in preventing re-bleeding from fundal varices as the bleed is flow-related than pressure-related and is associated with improved survival (45).

### 3.8. Newer perspectives

EUS-guided glue injection with or without coiling is safe and effective in primary and secondary prophylaxis of gastric varices bleeds (46, 47). Recent studies suggest performing TIPSS with BRTO in patients with recurrent variceal bleeding and spontaneous portosystemic shunts (SPSS) to prevent HE (48). The feasibility and cost-effective analysis of such procedures require further evaluation.

## 4. Ascites

Ascites is the most common complication of cirrhosis, and PH develops in ~85% of the cases (49, 50). To differentiate from other causes of ascites, ascitic fluid analysis is recommended, including serum-ascites albumin gradient (SAAG). SAAG value ≥ 1.1 g/dL has 97% sensitivity for PH as a cause of ascites (51). As discussed earlier, hepatic resistance and PH result in backflow and accumulation of vasodilatory substances, which results in intrahepatic vasoconstriction and peripheral vasodilation, including splanchnic vasodilation, which results in hypoperfusion of the renal system, even when the patient is euvolemic or hypervolemic (52). This state of relative hypovolemia due to vasodilation results in the activation of the renin-angiotensin-aldosterone system (RAAS) and sympathetic nervous system (SNS), leading to salt and fluid retention (50). This leads to the retention of blood and a significant rise in blood volume leading to filtration from the liver surface and the mesenteric vessels. High hydrostatic pressure, low oncotic pressure (hypoalbuminemia), and increased vascular permeability contribute to increasing filtration through mesenteric vessels. The resorptive capacity of the peritoneum and lymphatics cannot counteract these mechanisms (53). Any inflammation or infection of the peritoneum can affect peritoneal resorption. Dysregulation of these can lead to an increase in ascitic fluid formation.

### 4.1. Management of ascites

The foremost important part of the treatment of ascites is sodium restriction (salt intake of < 5 g) and the judicious use of diuretics. A combination of two diuretic classes (aldosterone antagonists and loop diuretics) is better tolerated and more effective than sequential treatment (i.e., first aldosterone antagonists followed by loop diuretics) (54). Use of albumin replacement and increased oral protein intake helps ascites mobilization. A recent pilot study has shown that early use of midodrine for a short course can control ascites better than diuretics alone, with a lesser occurrence of diuretic complications (55).

**Figure 4:**
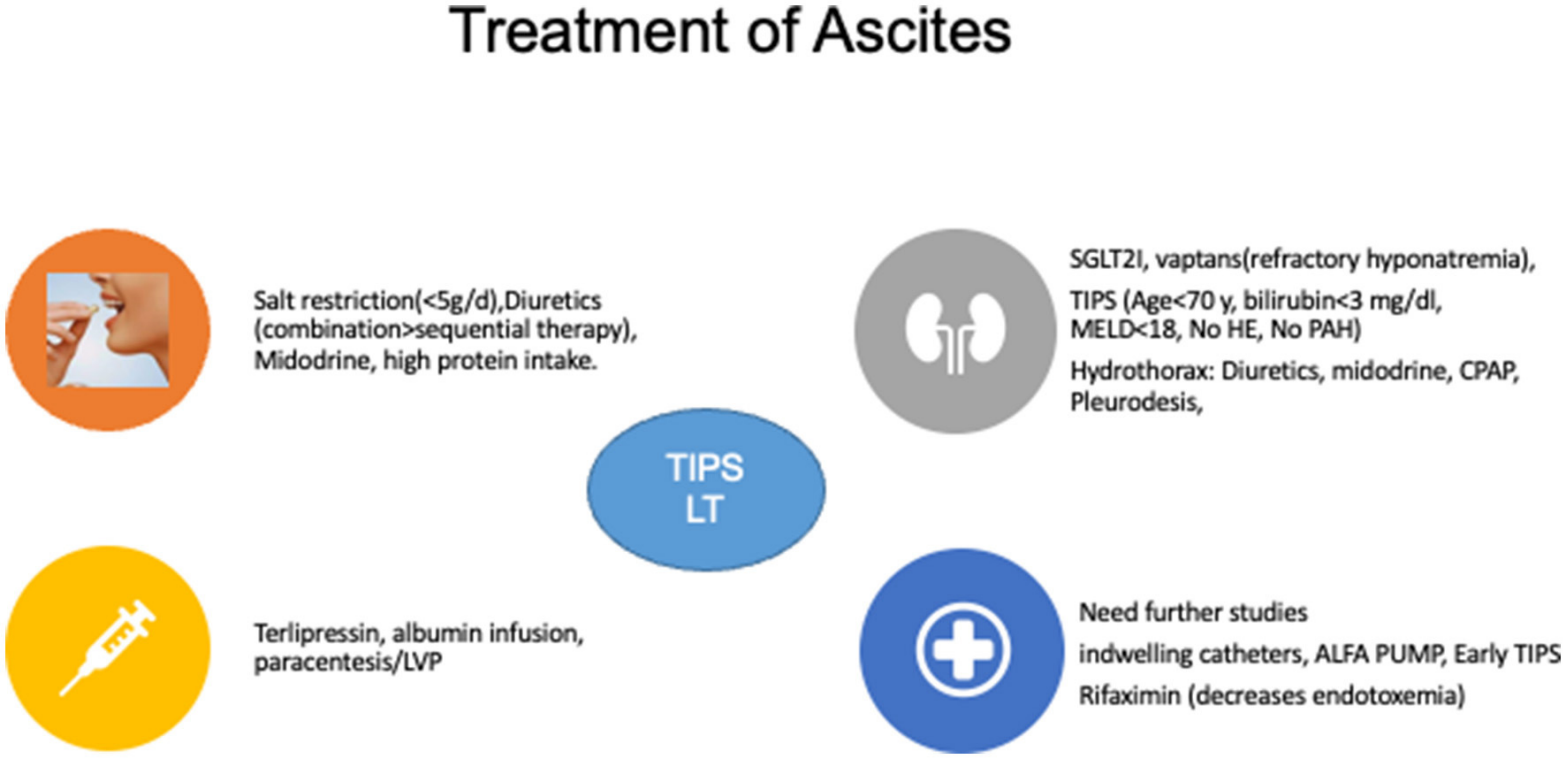
Treatment of ascites. SGLTA2I, Sodium-Glucose Co-transporter 2 inhibitors; MELD, model for end-stage liver disease; HE, hepatic encephalopathy; PAH, pulmonary arterial hypertension; TIPSS, transjugular intrahepatic portosystemic shunts; CPAP, continuous positive airway pressure; LVP, large volume paracentesis; LT, liver transplantation.

*RA:* A weight loss of < 0.8 kg over 4 days in a patient with cirrhosis on intensive diuretic therapy for at least 1 week is termed diuretic-resistant ascites, provided the urinary sodium is less than the sodium intake/day (56). Furosemide 160 mg/day and spironolactone 400 mg/day are considered for intensive diuretic therapy. Diuretic-resistant ascites is a rare event, especially in Asian countries, as the recommended full dose of diuretics (160 mg of furosemide and 400 mg of spironolactone) is rarely reached as most patients develop adverse events with higher recommended doses, which is called diuretic-intractable ascites (4, 55). This diuretic intolerance in the Asian population is due to a higher incidence of sarcopenia, poor muscle reserve, and a higher occurrence of diuretic-related complications, including renal injury and electrolyte imbalances (4). Before labeling a patient as refractory to therapy, hepatocellular carcinoma, portal vein thrombosis, and infection of the peritoneum [sepsis, spontaneous bacterial peritonitis (SBP)/Non-SBP/tuberculosis] should be ruled out. An elevated ascitic fluid protein content of more than 2–2.5 g/dl is suggestive of tuberculous ascites (57). Moreover, the higher incidence of tuberculosis in Asian countries can occur in immuno-compromised cirrhosis patients without manifesting classical signs and symptoms. Therefore, adenosine deaminase (ADA) and gene x-pert (tuberculosis nucleic acid testing) analysis of ascitic fluid is suggested in all patients with cirrhosis with difficulty uncontrolled ascites before labeling them as RA, especially in tuberculosis-endemic countries (4). LT is the best and ideal treatment option for patients with RA. Large-volume paracentesis (LVP > 5 L) with albumin infusion (8 gm/L of ascites removed) is the recommended therapy to relieve the symptoms. However, LVP is associated with the risk of paracentesis-induced circulatory dysfunction (PICD), which is mitigated with concomitant albumin usage. In a network meta-analysis, midodrine was reported as superior to albumin in preventing PICD (55, 58, 59). NSBBs are contraindicated in patients with RA requiring LVP due to compromised cardiac performance (60). Midodrine, an alpha-1 agonist, is beneficial in RA as it increases urine sodium loss and urinary volume (61, 62). By reducing endotoxemia, rifaximin may offer an additional benefit in RA (63). Tolvaptan is beneficial in ascites control with survival benefit (64). However, tolvaptan has a black box warning as it can cause or precipitate bleeding episodes by platelet aggregation inhibition and depleting vitamin-K-dependent clotting factors and has a risk of liver injury (65). Therefore, its use should be cautious and restrictive to patients of grade 3 ascites/RA with refractory hyponatremia and should be used for the shortest duration possible (51). Terlipressin, the most used drug in hepatorenal syndrome (HRS) and RA, helps in ascites control by mobilizing ascites and increasing renal perfusion, glomerular filtration rate (GFR), and urinary sodium excretion (66). Long-term albumin administration in patients with ascites improves survival, decreases hospitalization, and reduces overt HE, ascites, SBP, and non-SBP infections (67). TIPSS is a valuable therapy in RA and has been found to increase transplant-free survival (68). Careful selection of patients for TIPSS after a proper cardiac evaluation is recommended. A patient with age < 70 years with preserved liver function tests and low severity scores (MELD < 18 and Child score < 8) without any history of HE in the preceding 6 months are candidates suitable for TIPSS (Figure 4).

### 4.2. Newer perspectives

The automated low-flow ascites pump (ALFA) system, a novel device that transfers ascites from the peritoneal cavity to the urinary bladder, is effective in patients with RA (69). However, it is not universally available, complicated to use, and has higher adverse events; therefore, its use is currently limited (69). ANSWER trial reported the beneficial effects in terms of survival of long-term albumin infusions in patients with decompensated cirrhosis (70). Although results have been contradictory from two recent large trials, future research with more clearly defined selection criteria and endpoints may streamline the use of long-term albumin in ascites (70, 71). Sodium-glucose co-transporter 2 inhibitors (SGLT2I) increase sodium and glucose excretion in the urine and decrease renin secretion, showing significant improvement in ascites besides glycemic control in a few small studies (72, 73). Major side effect is an increased risk of urinary infections. Further prospective studies are needed in cirrhosis patients with RA for SGLT2I. Patients with RA and poor quality of life required long-term abdominal drains/catheters as a palliative measure. Although deemed to have an increased risk of infections, preliminary studies have shown good technical success and low rates of life-threatening infections providing options for home-based care (74, 75).

## 5. Renal dysfunction: acute kidney injury and hepatorenal syndrome

HRS, a functional renal failure, is a potentially reversible renal injury in patients with cirrhosis and ascites due to decreased renal blood flow (76). An increase in serum creatinine by ≥0.3 mg/dl within 48 h or an increase of >50% from baseline value with or without a decrease in urinary output < 0.5 ml/kg for >6 h in patients with cirrhosis and ascites in the absence of other evident cause for acute renal injuries such as proteinuria, shock, or nephrotoxins is termed HRS-AKI (76).

Recently, there has been a suggestion for a change in terminology, with previous terms like HRS-1 and HRS-2 being replaced by more physiologic HRS-AKI, HRS-acute kidney disease (AKD), and HRS-chronic kidney disease (CKD). The estimated incidence of HRS is around 18% at 1 year and 39% at 5 years and is associated with an inferior median survival of ≤ 3 months without a transplant (51, 56).

Although several medical management options remain in HRS, LT is the definitive therapy. Vasoconstrictors (terlipressin, octreotide in combination with midodrine and noradrenaline) and albumin infusion are the cornerstones of the treatment of HRS. The crux of HRS therapy still revolves around an attempt to rule out other causes (infections, glomerular disease, shock, and acute tubular necrosis) concomitant with volume expansion with albumin for 48 h followed by initiation of vasoconstrictors. Terlipressin remains the most effective vasoconstrictor, with an infusion strategy of administration associated with lesser adverse events (77, 78). Patients with HRS who have not responded to therapy and have persistently low GFR (i.e., < 25 ml/min) for more than 1.5 months and/or dialysis dependence are candidates for simultaneous liver-kidney transplantation (SLKT) (56). Recurrent episodes of HRS or renal insult lead to the development of HRS–CKD. The development of CKD in cirrhosis is a poor prognostic marker in both pre- and post-transplant settings (79). Risk factors of HRS-AKI progression to HRS-CKD are terlipressin non-response, high MELD score, albuminuria, recurrent AKI episodes, and high baseline serum cystatin (80). Management of HRS-CKD is unclear and needs further studies. Although treatment with terlipressin, diuretics in case of fluid overload, vaptans in case of hyponatremia, midodrine, and TIPSS with a high risk of HE are some options, SLKT is the definitive treatment (81, 82).

### 5.1. Newer perspectives

The use of TIPSS in patients with HRS-CKD has been recently shown to improve renal function with excellent control of ascites across stages of CKD (83). Recent studies suggest frailty as a predictor of HRS-AKI (84). It is unknown whether branched-chain amino acid (BCAA) supplementation reduces the development of HRS-AKI. With the approval of terlipressin in the US setting, exciting research is expected, with initial data advocating early initiation of terlipressin at lower grades of AKI being associated with improved survival (85).

## 6. Hepatic encephalopathy

HE is a neuropsychiatric manifestation related to severe liver disease. HE in a patient with acute liver failure is termed type A, while those related to shunts are termed type B, and those with cirrhosis are termed type C. HE is graded as per West-Haven criteria. HE can be covert [minimal HE (MHE) and Grade I HE], which needs to be identified with the help of specialized neuropsychological tests. Covert HE is reported among 80% of patients with advanced liver disease, while overt HE is reported among 40% (86). Overt HE can be new onset, episodic, with an interval between episodes of >6 months, or recurrent, where further episode occurs within 6 months. Persistent HE refers to an uncommon entity with no resolution of HE. Refractory HE (lack of response after treatment of precipitants and on treatment with lactulose and rifaximin for 48 h) is an uncommon but serious condition and requires active investigation into hidden precipitating events (i.e., portosystemic shunt) and requires alternative diagnosis to be ruled out (87). Important alternative causes include septic encephalopathy (23%), alcohol withdrawal, seizure, dyselectrolytemia, metabolic disorders, drugs/toxins (7%), intracranial structural lesions (5%), psychiatric disorders (1%), and multiple causes together (8%) (88).

### 6.1. Pathophysiology of HE and effect of ammonia

Alterations in neurotransmission and brain–blood barrier coupled with persistent neuroinflammation and oxidative stress, apart from GABA-ergic or benzodiazepine pathway abnormalities, lead to disruptions in brain energy and blood flow, causing HE. Disturbed ammonia metabolism is the central and most studied event in HE, with complex multimodality mechanisms. In brief, as liver failure progresses, concentrations of ammonia increase which exerts its systemic effects and neurotoxicity through multiple pathways, including astrocyte swelling, inflammation, oxidative stress, mitochondrial permeability alterations, alteration in energy kinetics, and membrane potential alterations (89). Despite this implicating pathophysiological basis, no direct correlation has been established between the severity of HE and ammonia concentrations. However, it is imperative to state that in the presence of a normal ammonia level, the diagnosis of HE is almost always an exclusion.

A venous ammonia level of >55 μmol/L is 47% sensitive and 78.3% specific to diagnose HE (90). Other studies have identified a blood ammonia level cutoff of >133 μg/dl as a diagnostic of HE. Arterial ammonia is an excellent surrogate marker for the severity of HE in ACLF in advanced stages, and an ammonia level above 140 μg/dl at baseline or at any time point in first week with grades III–IV HE serves as a poor prognostic marker for 28- and 90-day survival (91). Venous NH3 is more variable; therefore, arterial ammonia measurements are used (91, 92).

Spontaneous portosystemic shunts (SPSS) should be actively looked for, especially in recurrent/refractory HE and where liver diseases are not advanced (e.g., MELD < 15). SPSS shunts are noted in 10–20% of patients with cirrhosis and PH. SPSS is a “release valve,” a compensatory mechanism to reduce the portal pressure and bypass normal liver flow. More than 90% of patients with large SPSS have enlarged spleen, hepatic atrophy, and thrombocytopenia (93). Identification of these shunts is essential as these need to be ligated at the time of liver transplant, or else the patient can have persistent HE, even after liver transplant.

### 6.2. Management strategies in HE

Correct identification of the precipitant is the key to the management of HE. Non-absorbable disaccharidases (lactulose/lactitol) are the first-line therapy. Adding polyethylene glycol to non-absorbable disaccharidases leads to earlier, sustainable improvement in HE with survival benefits (94). Studies have shown a positive role of rifaximin and intravenous L-ornithine L-aspartate (LOLA) in overt HE management (95, 96).

Diet and calorie requirements must be met, especially for patients with altered mentation who cannot take orally. Adequate calories (35–45 kcal/kg/day) and protein (1–1.5 gm/kg/day) are essential to improve overall nutritional status. BCAA may be beneficial as they are metabolized in muscle and brain and promote protein synthesis, suppress protein catabolism, and act as gluconeogenesis substrates (97). Rifaximin is an oral antibiotic with minimal absorption (< 0.4%), broad-spectrum activity against enteric bacteria, excellent tolerability, no significant drug interactions, and no dose adjustment requirement in hepatic or renal dysfunction (98). The evidence for using rifaximin in HE needs close attention. The most robust evidence for rifaximin is as an add-on agent to lactulose in HE recurrence. However, high-quality evidence does not support its use as monotherapy for treating an episode of HE and direct comparative trials with non-absorbable disaccharides.

When used in conjunction with lactulose, rifaximin is effective in HE improvement, mortality reduction, and reduction in length of hospital stay (99). Zinc is a co-factor of urea cycle enzymes, and zinc deficiency has been reported to precipitate HE, thereby mandating the use of zinc supplements in HE (100). Although few studies have reported improvement in HE with probiotics, it is currently not FDA-approved (101).

### 6.3. Newer perspectives

Ammonia-lowering agents (Phenylacetate, Phenylbutyrate, and Sodium Benzoate) and drugs affecting neurotransmission (flumazenil and bromocriptine) have been reported to be effective but are rarely used. Recent trials have demonstrated the efficacy of L-ornithine L-aspartate in critically ill patients with HE (102, 103). CARTO/PARTO of SPSS is an excellent modality for patients with HE (104). The side effects of shunt occlusion include worsening of esophageal varices (19–46%), new onset varices in 6%, and new/worsening ascites in 14% of cases. Fecal microbiota transplantation (FMT) or intestinal microbiota transplantation is a feasible and safe option for patients with recurrent or persistent HE (105). By modulating the gut flora favorably, FMT restores the altered gut–liver–brain axis. The role of human albumin infusions in the management of HE has been controversial. However, in a recent randomized controlled trial, of outpatients with cirrhosis, prior HE, and current MHE, albumin infusions improved cognitive function and quality of life (106). Along similar lines, a systematic review indicates a possible beneficial effect of albumin in overt HE (107).

## 7. Hyper-dynamic circulation

As discussed earlier, an imbalance between vasodilators and vasoconstrictor occurs in PH and leads to hepatic vasoconstriction and peripheral vasodilation, which leads to hyperdynamic circulation, which is a very close mimic of the septic state. This is also known as “Hepsis” (108). In cirrhosis, immunological mechanisms are compromised, leading to a state of cirrhosis-associated immune dysfunction (CAID), predisposing patients with cirrhosis to the development of sepsis, which leads to an increase in pathogen-associated molecular patterns (PAMPs) and cytokines (tumor necrosis factor-α, interleukin-1β) and other vasodilators including nitric oxide. Consequently, a cycle of preferential splanchnic vasodilatation leading to the activation of vasoconstrictive systems along with central hypovolemia and cardiovascular dysfunction leads to a gradual development of the hyperdynamic syndrome and multiple organ dysfunctions (50, 109, 110). Treatment is targeted on these fundamental mechanisms. Still, so far, no single agent has been found to take care of all these aspects, and multimodality management addressing underlying pathophysiology is advocated.

### 7.1. Newer perspectives

Obeticholic acid (OCA) has been used in several liver diseases, including non-alcoholic steatohepatitis, primary biliary cholangitis, and primary sclerosing cholangitis (111). OCA has been reported to effectively reduce intrahepatic vascular resistance and improve PH in pre-clinical models (112). A recent study showed the beneficial effects of curcumin in cirrhotic rats with PH due to its antifibrotic, vasoactive, and anti-angiogenesis actions (113). Curcumin counteracts the hyperdynamic circulation of cirrhosis by inhibiting endothelial nitric oxide synthetase (eNOS) activation and reducing mesenteric angiogenesis by blocking the vascular endothelial growth factor (VEGF) pathway. However, the current evidence is too premature to recommend these drugs.

## 8. Cirrhotic cardiomyopathy

Hyperdynamic syndrome in patients with cirrhosis and PH leads to persistently activated compensatory mechanisms, activation of RAAS, and SNS, which results in tachycardia, increase in cardiac output, and reduction in systemic vascular resistance and MAP. This phenomenon, over time, results in cardiac dysfunction, described as “cirrhotic cardiomyopathy (CCM).” Altered contractile response to stress, abnormalities in electrophysiologic transmission, and diastolic dysfunction are the characteristic features of CCM in the absence of any evident cardiac disease (114). These can be in compensated form and result in symptoms only in case of stress (e.g., volume overload and post-TIPSS). Dyspnea and exertional fatigue due to pulmonary edema is the most common manifestation. Some other complications include overt heart failure, pulmonary hypertension, arrhythmias, pericardial effusion, and cardiac thrombus formation. The proposed pathophysiological mechanisms include aberrant beta-adrenergic signaling, increased endocannabinoid activity, alterations in Na^+^/Ca^2+^ exchanger, and the negative inotropic effect of nitric oxide and carbon monoxide (115, 116).

CCM is associated with an increased risk of complications (including RA, HRS, and impaired response to stressors), leading to poor quality of life, increased morbidity, and mortality. A targeted heart rate reduction using ivabradine can improve cardiac filling and output (114). CCM is potentially reversible with LT, provided other pathological diseases of cardia are ruled out (114). There have been some contradictory viewpoints about the effect of CCM on disease severity, with one study showing the lack of association of CCM with the severity of PH or liver dysfunction and age being the predominant determinant of CCM (117). Further studies resolve the contradictory observations that are required. Treatment of CCM is non-specific and supportive and rests on minimizing the treatment and interventions which can aggravate CCM (118). LT should be considered for well-optimized stable CCM patients and good performance status (119). Management of heart failure is similar to non-cirrhotic patients, including salt and fluid restriction, use of diuretics, and afterload reduction. Cardiac glycosides are not effective in cirrhotic patients (120). The studies on NSBB are conflicting. β-blocker can reduce prolonged QT intervals with some improvement in electromechanical uncoupling but with a reduction in cardiac output, which can be detrimental (121, 122).

### 8.1. Newer perspectives

Targeted heart rate reduction to improve cardiac filling and thereby improve the cardiac output with ivabradine can be tried in sinus rhythm patients (114). Potassium-Canrenoate can reduce the left ventricular wall thickness and left ventricular diastolic dysfunction (LVDD) in patients with Child A cirrhosis (122). These require further randomized controlled trials before universal recommendation.

## 9. ACLF

### 9.1. Basic pathophysiological mechanisms and clinical outcomes in ACLF

Decompensation in cirrhosis is a dynamic process, and patients can transition in the Child stage between A and C, depending upon the type and number of decompensation. Therefore, decompensation can be an index/first event or a recurrent event after recovery from the first event. In some cases, it becomes very severe to cause hepatic or extrahepatic organ failures/organ dysfunctions and is identified as ACLF, which heralds high short-term mortality of over 15% at 28 days with organ dysfunctions/organ failures (123). It is a state of dysregulated inflammation with a potential for reversibility, and it is different from acute liver failure and acutely decompensated cirrhosis (91, 124).

**Figure 5:**
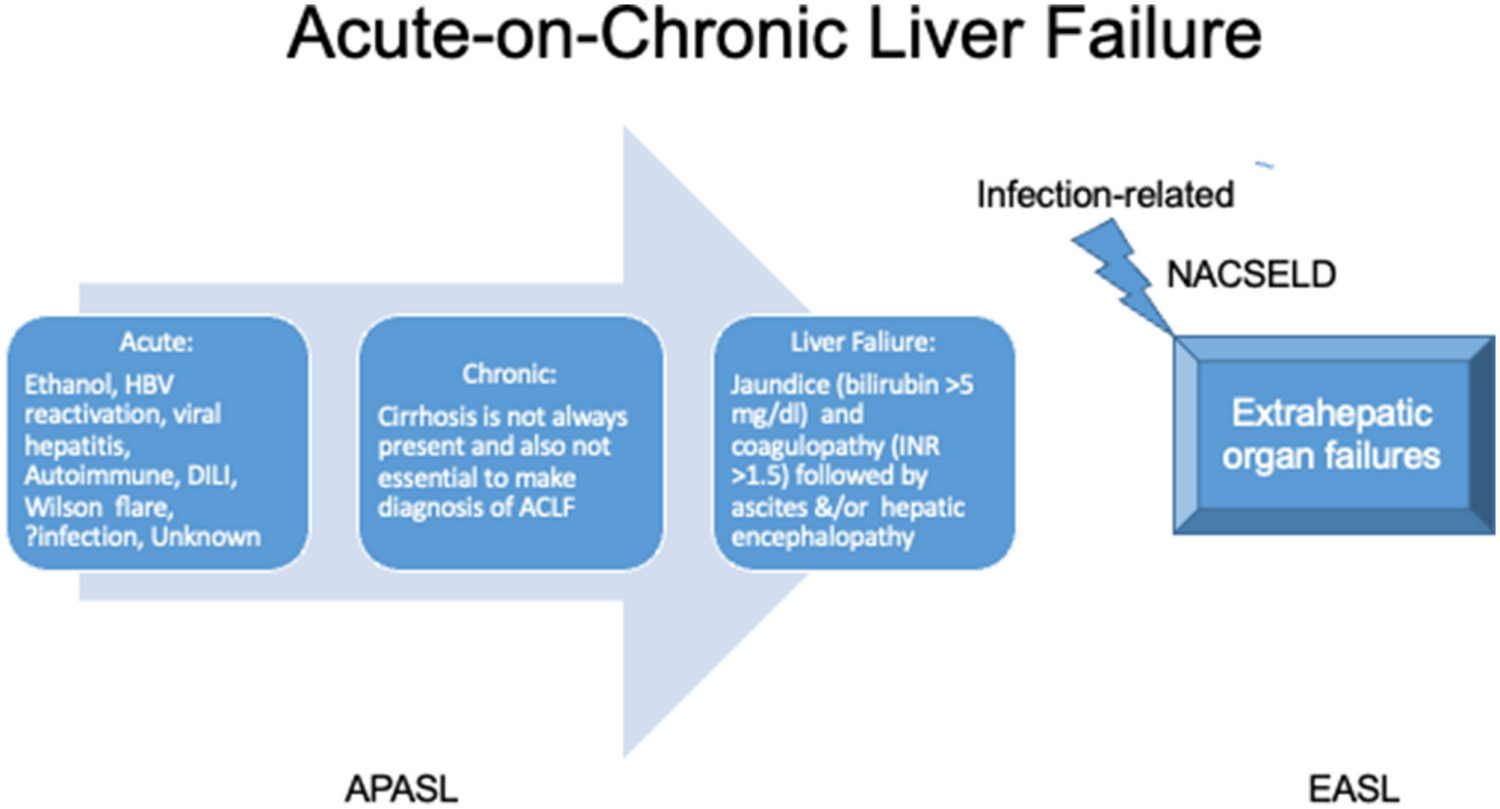
Definition of acute-on-chronic liver failure. APASL, Asian pacific association for the study of liver; EASL, European association for the study of the liver; NACSELD, North American Association for Study of Liver Diseases; INR, international normalized ratio; DILI, drug-induced liver injury.

Controversies exist between the definition and diagnostic criteria between east and west, but the central theme of the disease revolves around high short-term mortality (Figure 5). The two prominent definitions for ACLF are the Canonic by the European Association for the Study of Liver (EASL) and the Asian Pacific Association for Study of Liver (APASL) definition (Table 2). A large electronic database study reported significant discordance between APASL and EASL definitions (125). The incidence rate of ACLF as per APASL definition was 5.7 per 1,000 person-years, and the incidence rate of ACLF as per EASL definition was 20.1. Mortality was higher in EASL-identified ACLF than APASL identified (125). The median bilirubin level in the EASL-ACLF cohort was 2.0 mg/dL implying preserved liver function in EASL-ACLF. EASL and APASL criteria do not measure the same entity, and there is no uniformity in the ACLF definition. However, APASL ACLF is easier to use in clinical practice as it requires very few liver-specific laboratory variables (INR and bilirubin) and clinical history of ascites and/or encephalopathy.

**Table 2:** Differentiating two major definitions of acute on chronic liver failure (ACLF).

	**APASL-ACLF**	**EASL CLIF consortium**
Differences in key definition	Presence of an acute hepatic insult which manifests as jaundice with coagulopathy (INR > 1.5), and gets complicated within 4 weeks by ascites and/or encephalopathy in a patient with previously known or unknown chronic liver and has an intrinsically high 4-week mortality	Development of an acute deterioration of pre-existing chronic liver disease usually due to a precipitating event and leading to a high 4 week mortality due to multisystem organ failure.
Duration between insult and liver failure	4 weeks	Up to 12 weeks
What constitutes acute insult?	Only hepatic insults (alcoholic hepatitis, hepatotropic viruses, DILI, AIH)	Both hepatic and extrahepatic insults like infection and sepsis
How to define chronic liver disease?	Any chronic liver disease which is known or unknown which may or may not amount to cirrhosis (excludes previously decompensated cirrhosis)	Only patients with pre-defined cirrhosis including those with past history of decompensation
Sepsis	Is not considered as an acute event but may be a consequence of ACLF	A primary acute precipitant of liver failure and also may be a consequence
Variceal bleed as a precipitant	No consensus	Yes
Is reversibility defined?	Yes and is central to the definition	Not a clearly described
Disease severity score associated with definition	AARC	CLIF SOFA
Mortality described by definition	AARC-1:12.7%; AARC Grade 2:44.5% AARC Grade 3: 85.9%	ACLF Grade 1:22%; ACLF Grade 2: 33%; ACLF Grade 3:73%

APASL, Asia-Pacific Association for the Study of Liver; AARC, APASL-ACLF Research Consortium; CLIF, Chronic liver failure consortium; DILI, Drug-induced liver injury; AIH, Auto-immune hepatitis.

### 9.2. Key pathophysiological interplays in ACLF

**Figure 6:**
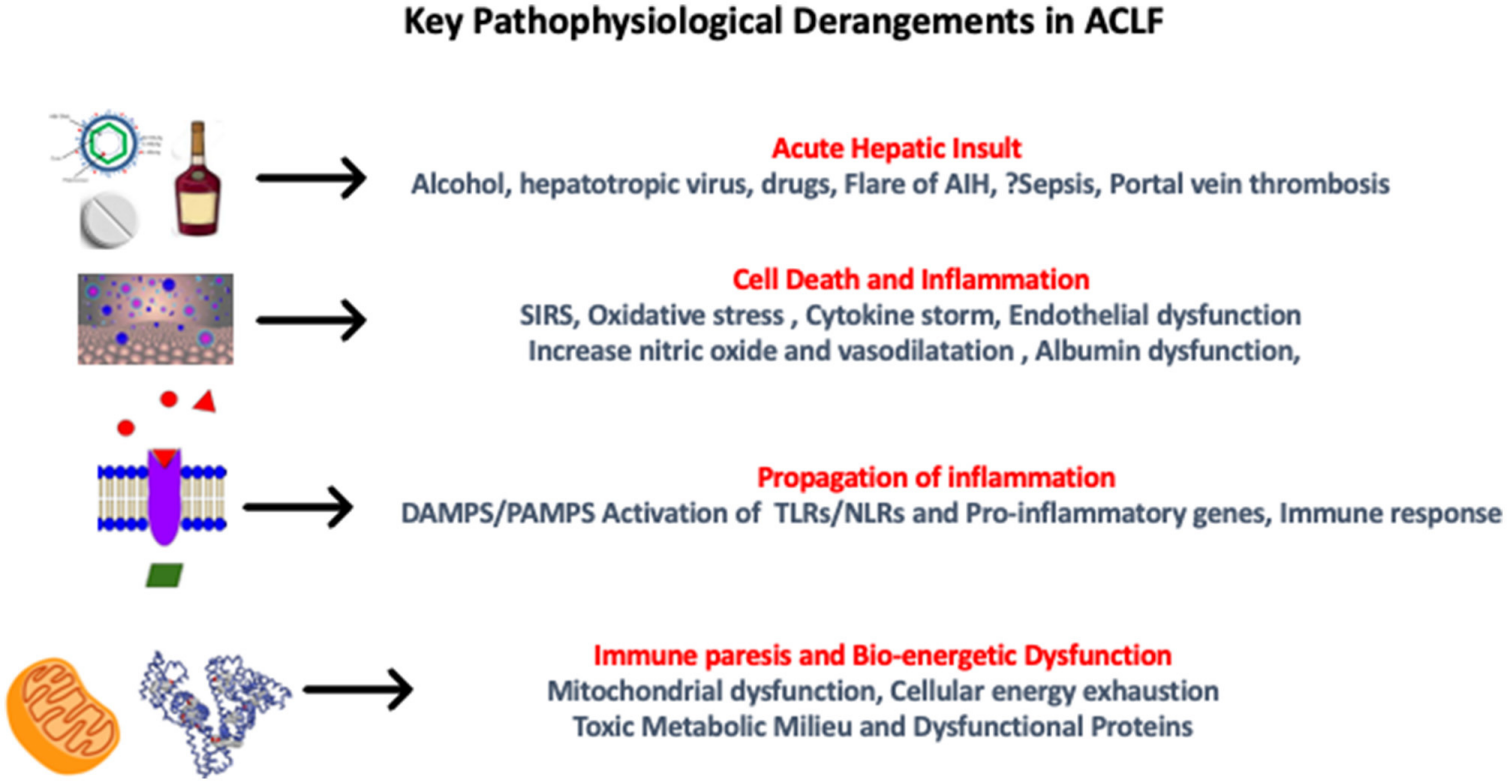
Pathophysiological derangements in acute-on-chronic liver failure. AIH, autoimmune hepatitis; SIRS, systemic inflammatory response syndrome; DAMPS, damage-associated molecular patterns; PAMPS, pathogen-associated molecular patterns; TLR, toll-like receptors; NLR, neutrophil-to-lymphocyte ratio.

Systemic Inflammatory Response Syndrome (SIRS) and sepsis are the keys to the development of ACLF, which is caused by gut dysbiosis, leaky gut, increased intestinal translocation of viable bacteria, and PAMPs (110). In the initial phases of cirrhosis, lamina propria is the predominant site of inflammation in the gut, where it is contained with localized vasodilation, but as the disease progresses, there is the involvement of deeper structures leading to a leaky gut. Inflammation becomes pronounced as bacterial translocation occurs, along with products of bacterial metabolism and damage-associated molecular patterns (DAMPs) from the diseased liver. These changes occur rapidly and mostly coincide with a burst of systemic inflammation, SIRS, which is usually triggered by a precipitating event (126). Prostaglandin (PG) E2 and PGE2-EP4 pathway-mediated monocyte dysfunction are the predominant factors for immunosuppression in ACLF and lead to inflammation-related mitochondrial dysfunction (127, 128). Therefore, the overall pathogenesis is characterized by an initial cytokine burst presenting as SIRS, progression to compensatory anti-inflammatory response system (CARS), and associated immune paralysis, which leads to sepsis and multi-organ failure (Figure 6).

### 9.3. ACLF and acute decompensation

Acute decompensation (AD) of chronic liver disease refers to a sudden worsening of the condition of a previously compensated or decompensated cirrhotic patient due to an acute event that may present with hepatic (jaundice, ascites, and HE) or non-hepatic (VH, AKI, or sepsis) failure, up to 3 months of acute insult (91). ACLF is a distinct syndrome from “AD” due to intense systemic inflammation in ACLF. The precipitant for AD can be hepatic or non-hepatic (129). Mortality in patients with AD (< 30% at 3 months) is lower than in those with ACLF (91). Management of AD and ACLF is quite similar, and LT would be the treatment of choice.

### 9.4. Precipitating events in ACLF

Since ACLF is triggered by an acute insult and has a potential for reversibility, identifying precipitating events is crucial so that targeted treatment can be instituted for better outcomes. Bacterial infections and active alcohol intake are the most common precipitating event in the west. In contrast, hepatitis B reactivation, followed by active sepsis and alcohol intake, is the most frequent precipitating event in the eastern world. However, no precipitating event may be found in about 40% of cases (129). In Asia, 1.8–5.7% of precipitating events are drugs related, which present as drug-induced liver injury (DILI) (130, 131). Acute viral hepatitis like hepatitis A, E, and other hepatotropic viruses can cause AD in ACLF. In addition, the flare of autoimmune hepatitis (AIH) can frequently be the precipitating event in female patients. Patients with AIH-related ACLF present histological features typical of AIH, including perivenulitis, lymphoid aggregates, and massive hepatic necrosis (132). The development of VH in patients with ACLF is an independent predictor of mortality (133). Acute hepatic venous outflow tract obstruction (HVOTO) or PVT can present as ACLF as per APASL guidelines (91). The underlying etiology of cirrhosis needs to be established in patients with ACLF presenting for the first time for appropriate management and prognostication.

### 9.5. Grade of ACLF

Organ failure (OF) includes both liver and extrahepatic organs. OF/organ dysfunction is the diagnostic hallmark of ACLF. CLIF-EASL grade is defined based on OF. *Grade-1* ACLF: only organ failure (renal, liver, coagulation, circulatory, or lung) that is associated with a serum creatinine level of 1.5–1.9 mg/dL; *Grade-2* ACLF: a combination of any 2 OFs. *Grade-3* ACLF: a combination of any 3 or more OFs (134). Conversely, the APASL definition is based on a dynamic score calculation known as the AARC score (91). AARC score between 5 and 7 is considered as APASL ACLF grade-1; 8–10 as AARC-2; and those with scores between 11 and 15 are AARC grade-3. Prognosis between the grades varies significantly, with grade 1 being a potentially recoverable group with a 28-day mortality of only 12.7%, and grade 3 needs immediate interventions to improve outcomes, with mortality at 28 days at around 85.9%.

### 9.6. SIRS, sepsis, ACLF, and LT

Liver failure predisposes to infections, and bacterial infections remain the most common cause of diseases in ACLF (135, 136). Infections are associated with severe inflammatory storms, high morbidity, cost, poor clinical course, and 4-fold high mortality (137). Sepsis is more likely associated with concomitant multi-organ involvement and poor prognosis (137). Frequency of infections in hospitalized cirrhotic patients ranges from 32 to 34% and increases with hospitalized cirrhotic patients with GI bleeding to 45%. The most common sites of first infections are SBP in 22–25%, urinary tract infection (UTI) in 20–28%, and pneumonia in 8–15% (137–139). Among pathogens, gram-negative (*E. coli* and *Klebsiella* spp.) are most frequent, followed by gram-positive (*Streptococcus pneumonia* and *Staphylococcus aureus*) and fungi (135).

Sepsis is an exaggerated inflammatory response to infection. SIRS in a patient with infection was required to identify sepsis (140). It is challenging to differentiate SIRS from sepsis due to the pre-existing hyperdynamic circulation in patients with cirrhosis and ACLF. Sepsis-3 criteria (rise of sofa score by 2 points) has been reported to be accurate in identifying sepsis in patients with cirrhosis. Furthermore, recent studies have suggested using fever and qSOFA scores to identify sepsis at the bedside (110, 141, 142). LT is the definitive therapy for ACLF. Early identification of those requiring LT or those who will have the resolution is the key to prolonging the survival of a patient with ACLF. Most hospitalized patients with ACLF have a clear prognosis between 3 and 7 days in either direction (143). Therefore, the concept of a transplant window period has been proposed by APASL and EASL (143, 144). Although early LT is associated with improved survival, such strategies are difficult in Asian settings where living donor liver transplantation is frequent, and the acceptance of LT is poor (145). In a large multination study of more than 1,000 patients who required LT, only 4% underwent LT (144).

### 9.7. Mechanisms of infections and organ failure

Damaged hepatocytes in liver diseases become dysfunctional and cause impaired protein synthesis, which leads to immune dysfunction. Disruption of gut homeostasis with altered gut permeability increases the translocation of bacterial products, and persistent low-grade inflammation leads to non-response of the immune cells leading to immune exhaustion (146). Hepatocyte damage generates more DAMPs and PAMPs, which activate pattern recognition receptors and cytokine burst and hepatocyte death (147). OF results from simultaneous ongoing processes such as immune dysfunction, hemodynamic derangement, excessive CARS, and the exhaustion and dysfunction of critical innate and adaptive immune system cells. According to previous studies, one of the theories advocates both pro-inflammatory and anti-inflammatory responses occurring early and simultaneously, manifesting initially by an early, dominant, hyperinflammatory phase of fever, shock, and hypermetabolism, which then evolves over several days into a more protracted immunosuppressive late stage (148, 149). According to the second theory, there is an upregulation of genes of the innate immune response and a downregulation of genes of the adaptive immune response, leading to inflammation driven by the innate immune system with resultant organ dysfunction and failure (150).

### 9.8. Management options in ACLF

Nutritional rehabilitation is one of the cornerstones of the management of ACLF. A target of 1.5–2.0 g protein/kg per day and 35–40 kcal/kg per day with carbohydrate-predominant late-evening snacks is recommended for patients with advanced cirrhosis. Regular screening and clinical examination of patients with ACLF may help identify the infection and organ failures early. Antibiotics should be part of ACLF management irrespective of sepsis/SIRS status due to the high risk of infection-related complications, which can mimic liver failure. Albumin infusions can prevent organ dysfunction in patients with SBP. However, the evidence to support its use in non-SBP infections and ACLF is limited. Terlipressin and albumin have been demonstrated to be beneficial in patients with ACLF (151, 152). FDA has recently approved terlipressin for HRS-AKI but has restricted its use in patients with ACLF-grade 3 due to the risk of pulmonary overload and ischemic adverse events (152–154). Specific treatments are available as antiviral strategies in HBV reactivation, steroids for severe alcoholic hepatitis and AIH, withdrawal of offending drugs for DILI, and chelators and plasma exchange (PE) for Wilson's disease. PE has been shown to improve systemic inflammation and reduce OF development in ACLF (155). It offers significant survival benefits over other liver support systems and could be a preferred modality of liver support for ACLF patients. FMT is safe in a small study and was associated with improved short-term and medium-term survival of alcohol-related ACLF (156). LT has been shown to have excellent results in ACLF except in patients with high grades of respiratory or circulatory failure (157). The survival benefits of LT in ACLF have been shown convincingly in a large systematic review involving 22238 LT recipients, with worse outcomes only being reported in the subgroup of ACLF 3 when compared to 30791 non-ACLF recipients (158). The grade of ACLF on days 3–7 determines the outcomes of patients, and as such, patients, particularly those with advanced grades, merit early transplant consideration and listing of potentially viable candidates (143). However, such an early listing (< 7 days) is impractical in resource-limited settings, while LT remains the therapy of choice as non-transplanted patients with ACLF have dismal survival of 8% at 1 year compared to 80% in those who undergo LT (159). Determination of timely access to LT facilities within the “window to transplant” is essential, beyond which LT is possibly a futile effort. Several areas need further research, including uniformity in definition and non-transplant measures to improve outcomes. Identifying futility is an important aspect of listing ACLF patients for LT. Some of the indicators of futility include patients with ≥4 organ failures, CLIF-C score > 64 at day 3–7, ACLF grade 2/3 patients with either active GI bleed, controlled sepsis for < 24 h, high vasopressor support (3 mg/h), PaO2/FiO2 (P/F) ratio < 150, active drug abuse; infections by MDROs or invasive fungal infections, high cardiac risk, and significant comorbidities (143, 159–161).

### 9.9. Newer perspectives

#### 9.9.1. Acute event and ACLF

Since the central concept of ACLF revolves around acute precipitation, the identification of acute precipitants is of key importance in the management of ACLF. There remain differences between the east and the west regarding the type of precipitants, with bacterial infections being the most common in the West while alcohol and hepatotropic viruses are common in Asia. In ~2–16% of the patients, no precipitant is identified (162). In this context, there has been recent interest in the identification of uncommon precipitants like cytomegalovirus as potential acute precipitants in ACLF in the background of a state of immune dysfunction in ACLF with CMV positivity in up to 24% of the cases (163). Similarly, drug-induced liver injury has been more frequently recognized with a large cohort of 3,132 patients with ACLF, having DILI as the precipitating event in 10.5% out of which the most common were complementary and alternative medications (71.7%) (164). However, therapeutic treatment of DILI is elusive and serves as an important area for future research. Recently, coronavirus disease (COVID-19) has been added to the list of precipitants of ACLF, which can be modified by vaccination (165–169). Surgical interventions (hepatic and non-hepatic) have also been investigated as precipitants of ACLF, with 24.5% developing ACLF in a cohort of 369 patients, with potential determinants being advanced age, hyponatremia, baseline bacterial infection, and abdominal non-hepatic surgery (170). Patients undergoing TIPSS, if sarcopenic, are at an increased risk of developing ACLF and consequent increased risk of hepatic encephalopathy and mortality (171). Interestingly, surgical interventions in patients who already have ACLF has also been studied and propensity-matched against TIPSS, with elective surgery being an independent predictor of worse outcomes and a recommendation to avoid elective surgery in those with ACLF and CLIF-C AD score of ≥50 (172).

#### 9.9.2. Sarcopenia and ACLF

The impact of sarcopenia as an independent predictor for mortality in patients with decompensated cirrhosis has been well-studied. The reported prevalence of sarcopenia in ACLF based on CT skeletal muscle index is 55.6% but was not found to be an independent predictor of mortality after adjusting for inherent liver dysfunction (173). However, it is important to note that based on a preliminary retrospective analysis, sarcopenia appears to co-relate with the severity or grade of ACLF as well as is an important predictor of post-transplant 1-year survival (174). Use of novel bedside methods of sarcopenia assessment, like muscle ultrasound techniques in this critically ill cohort of ACLF, appears a promising research subject (175).

#### 9.9.3. Therapeutics, transplantation, and ACLF

Even a modest volume of paracentesis (< 5 L) is associated with an increased risk of PICD in patients with ACLF, wherein midodrine is comparable to albumin in preventing PICD (176). While prophylaxis with norfloxacin effectively prevents infection in recovering patients of ACLF, a combination of low-dose corticosteroids with low-volume PE has been shown to improve short-term survival in ACLF in a small trial (135, 177).

Identification of prognostic models for predicting outcomes for LT in ACLF, especially in those with the highest grade of ACLF, is the need of the hour. Mortality prediction systems are central to ACLF, with artificial intelligence-based models being shown to be better than standard prognostic scores (178). A simplified prognostic model comprising age, pretransplant arterial lactate, leucocyte count, and respiratory failure and referred to as the TAM model (transplantation for ACLF-3 model) has been proposed. The model classifies a cutoff at 2 points to distinguish between a high-risk group (score > 2) and a low-risk group (score ≤ 2) with a 1-year survival of 8.3 vs. 83.9%, respectively (179). The score has been further validated to stress the importance of downstaging and stabilizing patients with ACLF before transplant, with those with a downstaged favorable TAM score having a significantly higher post-LT survival rate than those with static or incremental TAM score (88 vs. 70%) (180). Despite evolving data on the success of LT in ACLF, there remain variations and inequalities in both prioritization and access to LT in this subgroup which calls for increasing interdisciplinary interactions and awareness (181). Establishing a balance adjusting for the success of LT and resource utilization is imperative as LT in ACLF has also been shown to be highly resource-consuming with regard to healthcare use and costs (182).

#### 9.9.4. Prevention of ACLF and recompensation in ACLF

The field of ACLF has seen rapid developments and a plethora of research in the recent past. On the preventive aspect, exposure to statins and a decrease in von Willebrand factor (after NSBB therapy) have been shown to prevent subsequent ACLF development (183, 184). Rifaximin, in a recent retrospective study, has been shown to reduce clinical complications and progression to ACLF in patients with severe AH (185). Sepsis is a common precipitant of ACLF through the LPS-TLR4 pathway (186). Recombinant alkaline phosphatase (recAP), may reduce the risk of organ dysfunction by dephosphorylating the endotoxins and containing hepatic TLR4 expression (186, 187). Resatorvid (TAK-242) is a small-molecule inhibitor of TLR4 and is being utilized for the prevention of organ failures. Yak-001, an orally administered, non-absorbable, synthetic microporous carbon, has a high adsorptive capacity for bacterial products, lipopolysaccharides, and pro-inflammatory cytokines. Yak-001 was found to be safe and effective in reducing endotoxemia and inflammatory mediators (188). DIALIVE, a novel liver dialysis device that replaces dysfunctional albumin and removes pathogen-associated and damage-associated molecular patterns, has been shown to improve outcomes in patients with ACLF (189, 190).

## 10. Conclusion

Early identification of the severity of PH and addressing downstream complications is central to the management of cirrhosis. Each complication merits detailed redressal, and overall management demands a holistic approach. ACLF needs to be identified early in the course with the institution of specific therapies. Newer modalities such as plasmapheresis and FMT have promising results. LT remains the definitive care in both advanced cirrhosis and ACLF.

## Author contributions

AK and RJ made the study design and concept. RJ, AK, and AR prepared the initial draft. Figures by MP and KK. MS, PR, and DR provided the technical support. AK and MP critically reviewed and edited the final manuscript. All authors approved the final version.
